# Timosaponin AⅢ induces drug-metabolizing enzymes by activating constitutive androstane receptor (CAR) *via* dephosphorylation of the EGFR signaling pathway

**DOI:** 10.7555/JBR.38.20240055

**Published:** 2024-05-31

**Authors:** Muhammad Zubair Hafiz, Jie Pan, Zhiwei Gao, Ying Huo, Haobin Wang, Wei Liu, Jian Yang

**Affiliations:** Department of Pharmacology, Nanjing Medical University, Nanjing, Jiangsu 211166, China

**Keywords:** timosaponin AⅢ, CAR, metabolism enzyme, ERK1/2 signaling pathway, EGFR signaling pathway

## Abstract

The current study aimed to assess the effect of timosaponin AⅢ (T-AⅢ) on drug-metabolizing enzymes during anticancer therapy. The *in vivo* experiments were conducted on nude and ICR mice. Following a 24-day administration of T-AⅢ, the nude mice exhibited an induction of CYP2B10, MDR1, and CYP3A11 expression in the liver tissues. In the ICR mice, the expression levels of CYP2B10 and MDR1 increased after a three-day T-AⅢ administration. The *in vitro* assessments with HepG2 cells revealed that T-AⅢ induced the expression of CYP2B6, MDR1, and CYP3A4, along with constitutive androstane receptor (CAR) activation. Treatment with *CAR* siRNA reversed the T-AⅢ-induced increases in CYP2B6 and CYP3A4 expression. Furthermore, other CAR target genes also showed a significant increase in the expression. The up-regulation of murine CAR was observed in the liver tissues of both nude and ICR mice. Subsequent findings demonstrated that T-AⅢ activated CAR by inhibiting ERK1/2 phosphorylation, with this effect being partially reversed by the ERK activator t-BHQ. Inhibition of the ERK1/2 signaling pathway was also observed *in vivo*. Additionally, T-AⅢinhibited the phosphorylation of EGFR at Tyr1173 and Tyr845, and suppressed EGF-induced phosphorylation of EGFR, ERK, and CAR. In the nude mice, T-AⅢ also inhibited EGFR phosphorylation. These results collectively indicate that T-AⅢ is a novel CAR activator through inhibition of the EGFR pathway.

## Introduction

Timosaponin AⅢ (T-AⅢ) is a steroidal saponin derived from the *Anemarrhena asphodeloides* Bunge plant. It has reportedly possessed anti-platelet aggregation, anti-inflammatory, anti-diabetic, anti-depressant, and antitumor effects^[1]^. Several studies have highlighted the crucial role of T-AⅢ in inhibiting the growth of various cancer cells by disrupting the cell cycle^[2]^, inducing apoptosis, inhibiting invasion and migration^[3]^, suppressing angiogenesis^[4]^, and inducing ferroptosis^[5]^. These findings underscore its potential as an anticancer agent. Therefore, it is necessary to clarify pharmacokinetic characteristics of T-AⅢ, especially its effect on drug-metabolizing enzymes, which represents a common cause of hazardous drug-drug interactions. However, currently, the reports of T-AⅢin this area are minimal. Our preliminary results showed that T-AⅢ up-regulated the expression of the nuclear receptor, constitutive androstane receptor (CAR; NR1I3) in the liver of mice.

CAR serves as a master regulator of drug metabolism and disposition. It also plays a pivotal role in the development of various diseases, including cancers, liver diseases, inflammatory diseases, and metabolic disorders, through the regulation of energy homeostasis and cell proliferation^[6]^. Existing evidence suggests that CAR is a promising target for drug discovery, leading to extensive efforts to identify activators and inhibitors of CAR^[7]^.

As a transcription factor, CAR plays a crucial role in the regulation of drug metabolism by modulating the transcription of numerous target genes, including *CYP2B6*^[8]^, *CYP3A4*^[9]^, *CYP2C9*^[10]^, *CYP2A6*^[11]^, *CYP1A1*^[12]^, and *CYP1A2*. These genes collectively contribute to the metabolism of approximately 75% of clinically used drugs and the detoxification of numerous environmental chemicals, influencing drug safety and the risk of potential drug-drug interactions. The target genes of CAR also include isoforms of UDP-glucuronosyltransferases (UGTs)^[13]^, glutathione S-transferases (GSTs)^[14]^, sulfotransferases (SULTs)^[15]^, and multidrug resistance protein 1 (MDR1)^[16]^.

CAR exhibits unique properties distinct from prototypical nuclear receptors, involving both ligand-binding domain (LBD)-dependent (direct) and LBD-independent (indirect) activation mechanisms^[7]^. CAR consists of a common N-terminal activation function 1 ligand-independent domain, a conserved DNA-binding domain, and a C-terminal LBD^[17]^. Additionally, CAR may interact with co-activators/co-repressors, contingent on ligand-receptor binding. The binding of agonistic ligands induces conformational changes in the receptor, exposing the hydrophobic surface within the LBD for co-activator binding. Conversely, antagonistic ligands prompt co-repressor binding, leading to receptor deactivation^[18]^.

CAR remains inactive in the cytoplasm of hepatocytes as a homodimer phosphorylated at Thr38 within the DNA-binding domain. In this state, CAR physically interacts with extracellular signal-regulated protein kinases 1 and 2 (ERK1/2) near the C-terminus of CAR. This interaction allows CAR to form its homodimer and prevents CAR from binding to protein phosphatase 2Ac (PP2Ac), thereby maintaining CAR phosphorylation and cytoplasmic localization. The indirect activation of CAR ligands or the inhibition of ERK1/2 results in the dissociation of CAR homodimers into monomers. This process leads to the translocation of non-phosphorylated CAR monomers into the nucleus, where they interact with regulatory promoter regions as heterodimers with retinoid X receptor^[19]^, significantly inducing the expression of its classic target, *CYP2B10* mRNA, in primary mouse hepatocytes^[20]^. Phenobarbital is a typical indirect activator of CAR^[21]^.

The current study aimed to examine the effects and mechanisms of T-AⅢ as an anticancer agent on CAR and the corresponding drug-metabolizing enzymes.

## Materials and methods

### Materials

The isolation of T-AⅢ from *Anemarrhena asphodeloides* was carried out according to the established protocols^[2,22]^, conducted by Professor Huang of China Pharmaceutical University. T-AⅢ, with a purity exceeding 98%, was dissolved in dimethylsulfoxide (DMSO) to create a 10 mmol/L stock solution, which was stored at –20 ℃.

Antibodies for CYP2B6 (1∶1000, Cat. #ab69652), CYP3A4 (1∶2000, Cat. #ab3572), PXR (1 µg/mL, Cat. #ab118336), CAR (1∶1000, Cat. #ab186869), and MDR1 (P-gp) (1∶2000, Cat. #ab129450) were sourced from Abcam (Cambridge, MA, USA). The CYP2B10 antibody (1∶2000, Cat. #sc-73546) was acquired from Santa Cruz Biotechnology (Santa, TX, USA). Antibodies for ERK (1∶1000, Cat. #4695), p-ERK (1∶1000, Cat. #4511), AMPK (1∶1000, Cat. #5831S), p-AMPK (1∶1000, Cat. #50081S), JNK (1∶1000, Cat. #9252), p-JNK (1∶1000, Cat. #4668), p38 (1∶1000, Cat. #8690), p-p38 (1∶1000, Cat. #4511), and Histone H3 (1∶2000, Cat. #4499S) were obtained from Cell Signaling Technology (Danvers, MA, USA). Antibodies for EGFR (1∶1000, Cat. #BS1533), p-EGFR (Y1173) (1∶1000, Cat. #BS94062), p-EGFR (Y845) (1∶1000, Cat. #BS6349), GAPDH (1∶8000, Cat. #MB9231), and β-actin (1∶10000, Cat. #AP0060) were procured from Bioworld (Bloomington, MN, USA). The P-CAR (Thr38; 1∶1000) antibody was sourced from ABclonal (Wuhan, China). Additionally, horseradish peroxidase (HRP)-conjugated AffiniPure goat anti-rabbit IgG (H+L; 1∶5000, Cat. #SA00001-2) and HRP-conjugated AffiniPure goat anti-mouse IgG (H+L; 1∶5000, Cat. #SA00001-1) were purchased from Proteintech Group, Inc. (Chicago, IL, USA).

### Cell culture

Human hepatocellular carcinoma cells (HepG2) were obtained from the Cell Bank of the Chinese Academy of Sciences (Shanghai, China). These cells were maintained as monolayers in Dulbecco's modified Eagle's medium (DMEM, Gibco, Grand Island, NY, USA) supplemented with 10% fetal bovine serum (Hyclone, Logan, Utah, USA), 100 U/mL penicillin, and 100 U/mL streptomycin. Cultures were maintained in a humidified environment with 5% CO_2_ at 37 ℃. For experiments, cells were seeded in a 6-well plate at a density of 3 × 10^5^ cells per well overnight. Subsequently, the cells were treated with specific concentrations of T-AⅢ in the serum-reduced medium (1% fetal bovine serum) for 24 h.

### Animal experiments

Female BALB/c-nude mice (5 to 6 weeks, 19 to 22 g, specific pathogen-free grade) were obtained from Cavens Experimental Animal Technology Co., Ltd. (Changzhou, China). The mice were housed in controlled environments with a 12-h light/dark cycle and provided with standard laboratory food and water *ad libitum*. For subcutaneous xenograft models, 150 μL of media containing 5 × 10^6^ MDA-MB-231 cells were injected subcutaneously into the right flanks of female BALB/c-nude mice. Once the tumor volume reached approximately 100 mm^3^ (measured using a digital caliper and the following formula: V = length × width^2^/2), the mice were randomly grouped into four groups, each comprising five mice. The groups were then treated every other day with vehicle (v/v; 1% Tween 80, 2% DMSO, 97% physiological saline), 2.5, 5, and 10 mg/kg body weight T-AⅢ *via* intraperitoneal injection, respectively. Weights of the mice and tumor volumes were recorded every other day. Mice were euthanized, when the volume of tumors in the vehicle-treated group reached 1000 mm^3^. Simultaneously, livers were excised and stored at −80 ℃ for subsequent Western blotting analysis.

Eight-week ICR mice were obtained from Jiangsu Laboratory Animal Center (Nanjing, Jiangsu, China). The mice were maintained under controlled environmental conditions (humidity, temperature) with a 12-h light/dark cycle, and allowed free access to water and diet. The mice were randomly assigned to four groups: one control group and three groups treated with different doses of T-AⅢ, each comprising five mice. Subsequently, the four groups of mice were treated with vehicle (v/v; 1% Tween 80, 2% DMSO, 97% physiological saline), 2.5, 5, and 10 mg/kg body weight T-AⅢ by intraperitoneal injection for consecutive three days, respectively. On the fourth day, the mice were sacrificed, and the livers were extracted and stored at –80 ℃ for subsequent Western blotting analysis. All procedures related to mice were strictly carried out according to ethical guidelines of the Animal Ethical and Welfare Committee of NJMU (Approval No. IACUC1911001).

### Colorimetric MTT assay

HepG2 cells were seeded at a density of 3 × 10^3^ cells per well in a 96-well plate overnight. The cells were then treated with different concentrations of T-AⅢ for 24 h. Subsequently, 10 µL of 3-(4,5-dimethylthiazol-2-yl)-2,5-diphenyltetrazolium bromide (MTT; 5 mg/mL in PBS) was added to each well, and the plate was incubated at 37 ℃ for an additional 4 h. After careful removal of the medium, 100 µL of dimethyl sulfoxide was added into each well to dissolve the precipitate. Finally, the absorbance was measured at 570 nm using an automated microplate reader ELx800 (Bio-Tek, Winooski, VT, USA). The cell survival ratio was expressed as a percentage of the control.

### Quantitative reverse transcription-PCR (qRT-PCR)

Total RNA was extracted from cells using Trizol following the manufacturer's instructions. The first-strand cDNA was synthesized from 1 µg of total RNA with the following cycling parameters: 25 ℃ for 10 min, 50 ℃ for 30 min, and 85 ℃ for 5 min, using supermix (Vazyme, Nanjing, Jiangsu, China). Quantitative real-time PCR was performed with SYBR Green Supermix (Bio-Rad, Hercules, CA, USA) using the Applied Biosystems QuantStudio 5 system (Bio-Rad). Changes in gene expression were calculated using the relative Ct method, and the values were normalized to the endogenous "housekeeping" gene *GAPDH* using the 2^−∆∆Ct^ method. The primer sequences used are listed in ***Table 1***.

**Table 1:** The primers for genes

Genes	Forward (5′-3′)	Reverse (5′-3′)
*CYP2B6*	GCACTCCTCACAGGACTCTTG	CCCAGGTGTACCGTGAAGAC
*MDR1*	GTCCCAGGAGCCCATCCT	CCCGGCTGTTGTCTCCATA
*CYP3A4*	TCAATAACAGTCTTTCCATTCCTCAT	CTTCGAGGCGACTTTCTTTCA
*CAR*	CAGGGTTCCAGTACGAGTT	AGCCGAGACTGTTGTTCC
*CYP1A1*	TCGGCCACGGAGTTTCTTC	GGTCAGCATGTGCCCAATCA
*CYYP1A2*	CTGGGCACTTCGACCCTTAC	TCTCATCGCTACTCTCAGGGA
*GSTK1*	TCTGGAAAAGATCGCAACGC	GCCCAAAGGCTCCGTATCTG
*GSTM1*	ATTGGCCTCCTGTATTCCTTGA	GTGCTCCGACAAATAGTCTGAAG
*SULT1A1*	GCCTTCTACGCCGGTATGAG	AGACCACCATATAGGTGTTCCA
*SULT1A2*	CCCCAGACTCTGTTGGATCAG	AACCGCCACATCCTTTGC
*UGT1A1*	CATGCTGGGAAGATACTGTTGAT	GCCCGAGACTAACAAAAGACTCT
*UGT1A6*	CTCCTTCGCTCATTTCAGAGAAT	CGGTCACTGAGAACCTCAACTAT
*UGT1A9*	CCCCCTTCCTCTATGTGTGTG	TCATACTCCGTAACAGGTGTTTG
*GAPDH*	GGAGCGAGATCCCTCCAAAAT	GGCTGTTGTCATACTTCTCATGG

### Immunofluorescence analysis

HepG2 cells were fixed with 2% paraformaldehyde in PBS (pH 7.4) at room temperature for 15 min followed by PBS washing and permeabilization with 0.1% Triton X-100 on ice for 15 min. Subsequently, cells were blocked with 2% BSA in PBS for 1 h and then incubated with an anti-CAR antibody at 4 ℃ overnight. After that, the fluorescein-5-isothiocyanate-conjugated AffiniPure goat anti-rabbit IgG (H+L) (1∶200; Cat. #BS10950, Bioworld) was added to cells for 1 h in a light-protected environment. For nuclear staining, cells were exposed to the DAPI staining kit (Louis Park, MN, USA) in the dark for 15 min. Finally, the cytosolic fluorescent intensity of CAR was examined under a confocal fluorescent microscope (Olympus, Tokyo, Japan).

### Preparation of cytosolic and nuclear extracts

HepG2 cells were treated with or without 2.5 μmol/L T-AⅢ for 12 h, and then were collected and nuclear extracts were prepared using a Nuclear Extract kit (Thermo Scientific, Rockford, IL, USA). Briefly, cells were lysed with CERⅠand vortexed vigorously on the highest setting for 15 s, then incubated on ice for 10 min. Ice-cold CERⅡ was added and vortexed on the highest setting for 5 s. The cells were centrifuged at 16000 *g* at 4 ℃ for 5 min, and the supernatants were saved as the cytosolic fractions. The nuclear pellets were resuspended in ice-cold NER and vortexed on the highest setting for 15 s every 10 min, totaling 40 min. After centrifugation at 16000 *g* under 4 ℃ for 5 min, the supernatants were saved as the nuclear fractions.

### MEK-ERK activator and inhibitor treatment

To elucidate the mechanism of T-AⅢ-induced CAR activation, tert-butylhydroquinone (t-BHQ; ERK activator, Macklin, Shanghai, China) and PD98059 (MEK inhibitor, Selleck, Houston, Texas, USA) were used. Cells were seeded at a density of 3 × 10^5^ cells per well in a 6-well plate and treated with t-BHQ (10 µmol/L) and PD98059 (10 µmol/L) 1 h before the addition of T-AⅢ (2.5 µmol/L). The cells were then incubated for an additional 24 h. Total protein was extracted from cells, and the expression levels of ERK, p-ERK, CAR, and its target genes were assessed.

### Western blotting analysis

Total protein was extracted from HepG2 or the liver of mice by using RIPA lysis buffer (Bioworld), and protein concentrations were determined with a BCA protein assay kit (Thermo Scientific) following the manufacturer's instructions. An equal concentration of protein was separated by SDS-polyacrylamide gel electrophoresis and transferred onto PVDF membranes (Bio-Rad) using a Bio-Rad Trans-Bolt Turbo transfer system (Bio-Rad). The membrane was blocked with 5% bovine serum albumin (BSA) in TBST for 2 h at room temperature followed by incubation with individual primary antibodies overnight at 4 ℃. After washing with TBST, the membrane was incubated with HRP-conjugated anti-mouse or anti-rabbit IgG (Bioworld) for 1 h. Immuno-reactive protein bands on the membrane were visualized using the ECL Western blotting detection system and captured on the Clinx Scanning System. Protein levels were quantified through density analysis with Image J software and expressed as the ratio of the desired protein/endogenous control (GAPDH or β-actin).

### Small interfering RNA (siRNA) transfection

siRNAs were custom-synthesized by Tsingke (Beijing, China) with the following sequences: human *CAR* #1, 5′‐GGAAUCUGUCACAUCGUA‐3′; human *CAR* #2, 5′‐CUCUGCAAAGCUACAUCAA‐3′; human *CAR* #3, 5′-CACACUUCGCAGACAUCAA‐3′. For transient transfection, cells were seeded on a 6-well plate at a density of 2.5 × 10^5^ cells/mL with antibiotic-free medium. After overnight incubation, the target siRNAs were transfected using GenJet transfection reagent (SignaGen, Maryland, USA) following the manufacturer's instructions.

### Statistical analysis

Data in the current study were presented as the mean ± standard deviation calculated from at least three replicates of experiments conducted in parallel. Statistical analyses were performed using an unpaired two-tailed Student's *t*-test or one-way analysis of variance (ANOVA) through GraphPad Prism6.

## Results

### T-AⅢ induced expression levels of CYP2B10, MDR1, and CYP3A11 in nude mice and ICR mice

**Figure 1:**
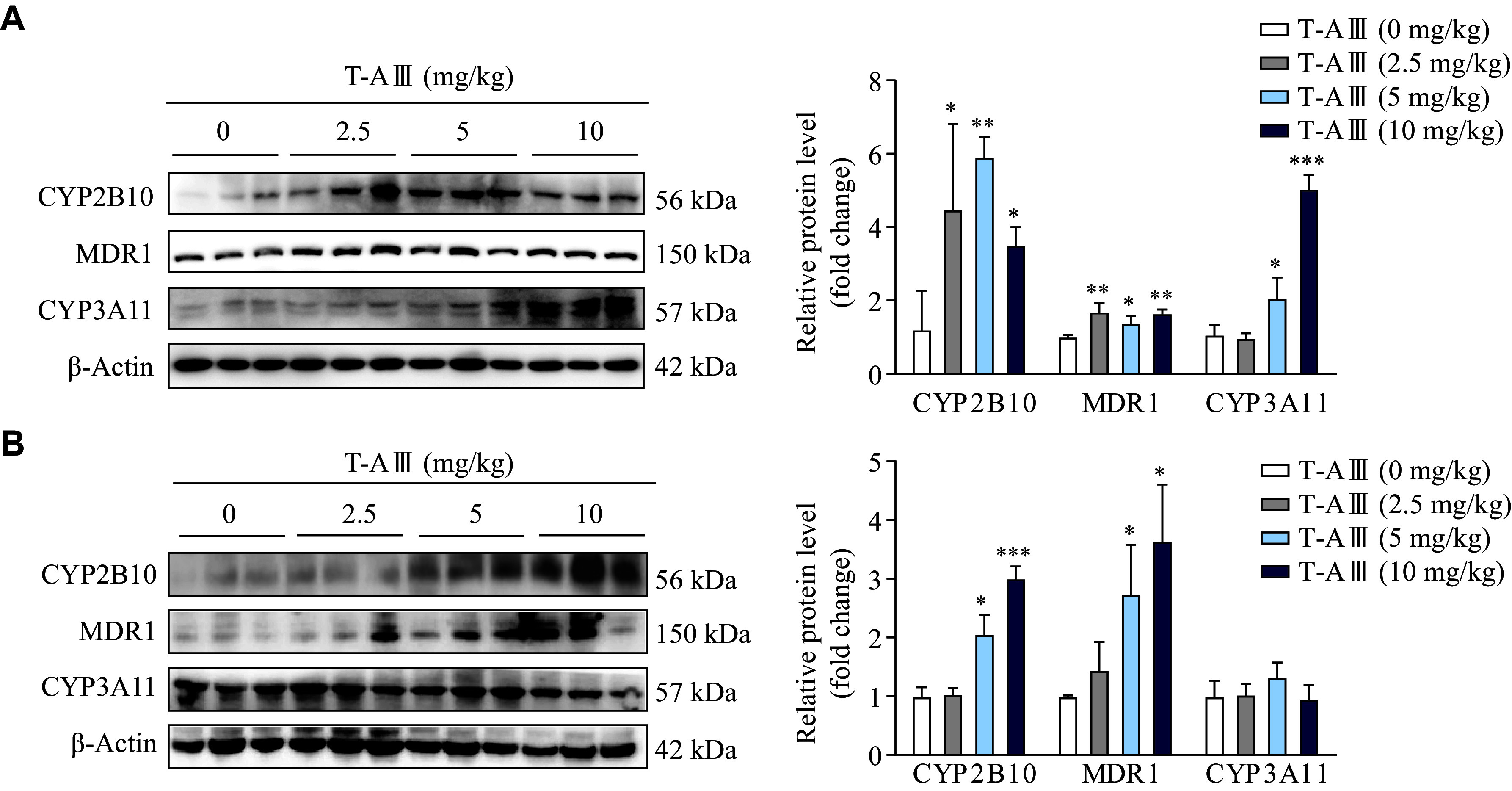
T-AⅢ induced the expression levels of CYP2B10, MDR1, and CYP3A11 in the liver tissues of both nude and ICR mice.

Our previous work demonstrated that T-AⅢ exerted an inhibitory effect on tumor growth in nude mice inoculated with MDA-MB-231 cells. Therefore, in the current study, we investigated the effects of T-AⅢ on drug-metabolizing enzymes when used in the treatment of breast cancer. MDA-MB-231 cells were injected subcutaneously into the right flanks of female BALB/c-nude mice to establish subcutaneous xenograft models. The nude mice were treated with various dosages of T-AⅢ (0, 2.5, 5, and 10 mg/kg body weight) for 24 days. The Western blotting analysis showed a significant increase in the expression levels of CYP2B10, MDR1, and CYP3A11 in the liver tissues of nude mice after T-AⅢ treatment (***Fig. 1A***). Meanwhile, we examined the effects of T-AⅢ in the ICR mice by treating them with various dosages of T-AⅢ (0, 2.5, 5, and 10 mg/kg body weight) for 3 days. The results showed that the 5 and 10 mg/kg T-AⅢ-treated groups exhibited a significant increase in the expression levels of CYP2B10 and MDR1, without any significant changes in the expression levels of CYP3A11 in the liver tissues of ICR mice (***Fig. 1B***). These findings suggest that T-AⅢ may induce the expression levels of CYP2B10, MDR1, and CYP3A11 when exerting anticancer effects, thereby accelerating the metabolism of corresponding substrates.

### T-AⅢ induced the expression levels of CYP2B6, MDR1, and CYP3A4 in HepG2 cells

**Figure 2:**
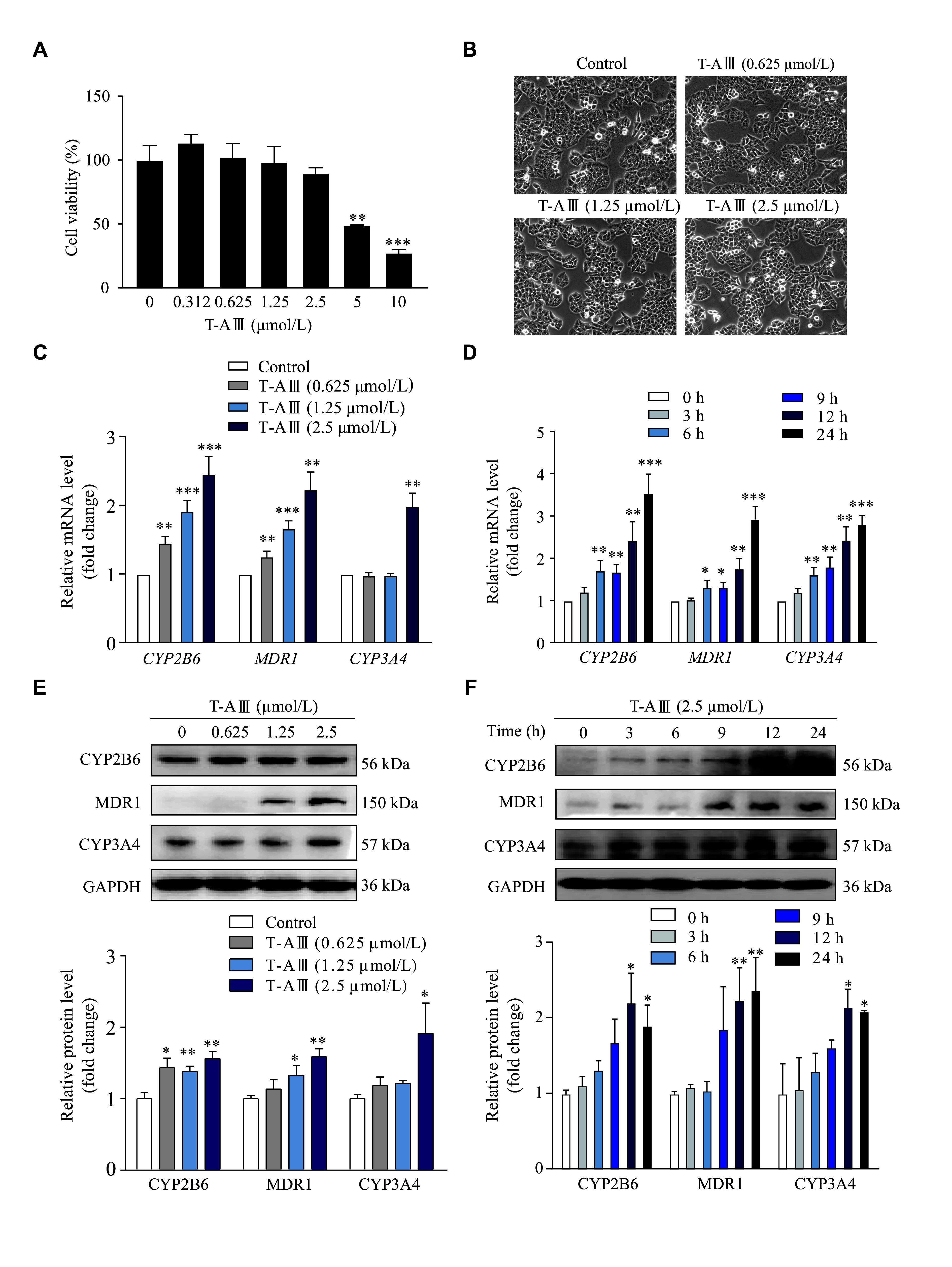
T-AⅢ induced the expression levels of CYP2B6, MDR1, and CYP3A4 in HepG2 cells.

We chose HepG2 cells to further validate the effect of T-AⅢ on metabolism enzymes. HepG2 cells were treated with different concentrations of T-AⅢ for 24 h, and the cell viability was analyzed by the MTT assay. The results showed that 5 and 10 µmol/L T-AⅢ exhibited significant cytotoxic activity against HepG2 cells (***Fig. 2A***). Furthermore, under the inverted light microscope, HepG2 cells treated with 0.625, 1.25, and 2.5 µmol/L of T-AⅢ for 24 h grew well (***Fig. 2B***). Based on these observations, 0.625, 1.25, and 2.5 µmol/L of T-AⅢ were selected for the subsequent investigations in the current study.

In HepG2 cells treated with different concentrations of T-AⅢ for 24 h, mRNA levels of *CYP2B6* and *MDR1* significantly increased in a concentration-dependent manner (***Fig. 2C***). Specifically, when treated with 2.5 μmol/L of T-AⅢ, *CYP2B6* and *MDR1* mRNA levels increased to 2.5- and 2.3-fold of the control, respectively. Whereas the mRNA levels of *CYP3A4* were increased to 2.0-fold only in 2.5 μmol/L of the T-AⅢ-treated cells.

In HepG2 cells treated with 2.5 μmol/L of T-AⅢ for various durations (0, 3, 6, 9, 12, and 24 h), the mRNA levels of *CYP2B6*, *MDR1*, and *CYP3A4* significantly increased after 6 h and then continuously amplified in a time-dependent manner (***Fig. 2D***). Meanwhile, the protein levels of CYP2B6, MDR1, and CYP3A4 enhanced in a concentration- and time-dependent manner, and the results matched the qRT-PCR results (***Fig. 2E*** and ***2F***). These results suggest that T-AⅢ induces expression levels of CYP2B6, MDR1, and CYP3A4 in HepG2 cells, consistent with that observed *in vivo*.

### T-AⅢ induced the expression and activation of nuclear receptor CAR *in vitro* and *in vivo*

**Figure 3:**
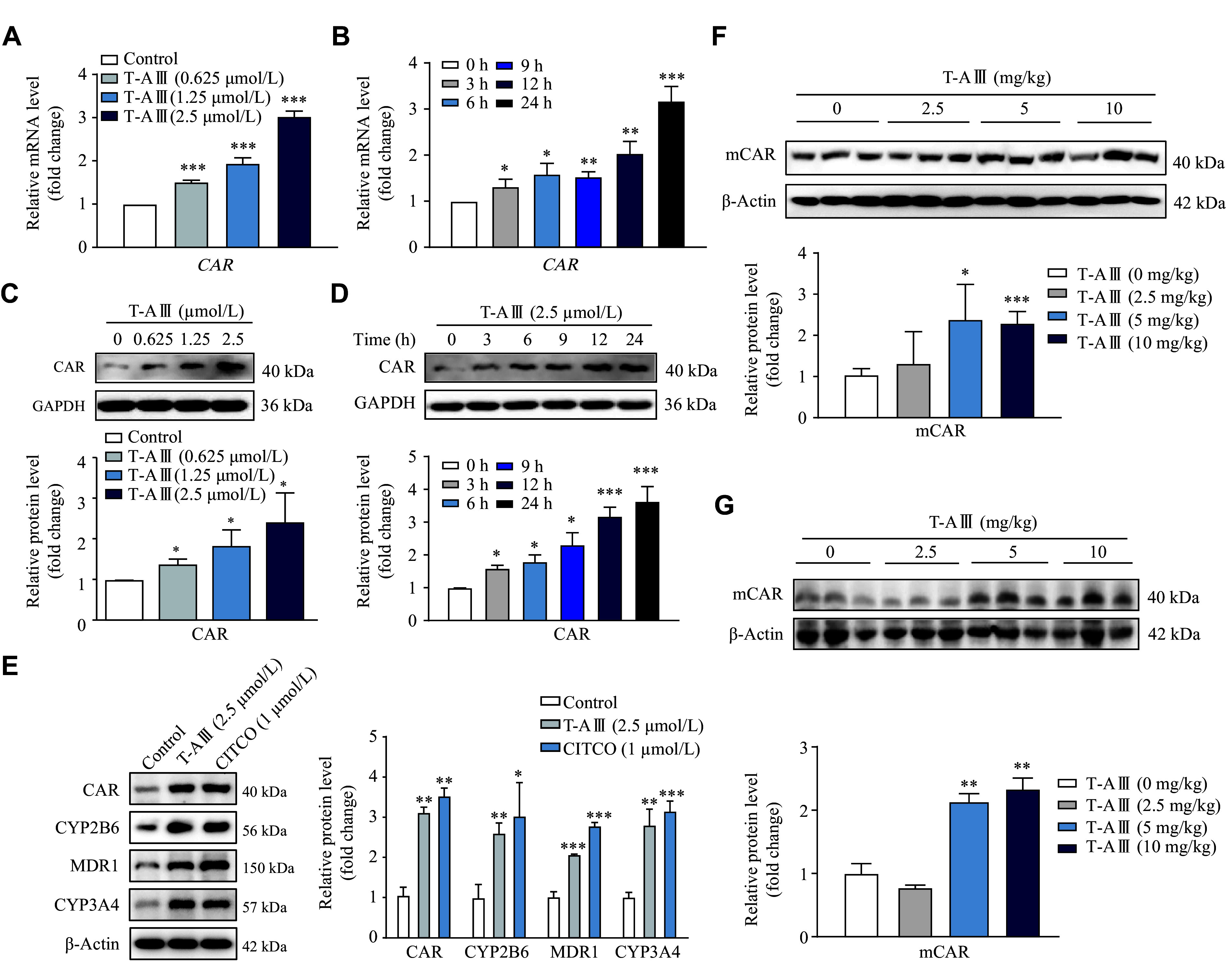
T-AⅢ induced nuclear receptor CAR expression *in vitro* and *in vivo*.

Nuclear receptor CAR plays a pivotal role in the regulation of transcriptional levels of *CYP2B6*, *CYP3A4*, and *MDR1*. Therefore, we evaluated the effects of T-AⅢ on CAR in HepG2 cells. The results showed that T-AⅢ significantly increased both mRNA and protein levels of CAR in a concentration-dependent manner (***Fig. 3A*** and ***3C***). Treatment with 2.5 μmol/L of T-AⅢ resulted in over two-fold increase in CAR mRNA and protein levels, compared with the control group. In HepG2 cells treated with 2.5 μmol/L of T-AⅢ for 0, 3, 6, 9, 12, and 24 h, significant increases in both mRNA and protein levels of CAR were observed 3 h after the treatment and then persistently improved in a time-dependent manner (***Fig. 3B*** and ***3D***). These results suggest that the T-AⅢ-mediated enhancement of expression levels of *CYP2B6*, *CYP3A4*, and *MDR1* may be regulated by CAR activation.

To further validate the induction effect of T-AⅢ on CAR, CITCO, a hCAR agonist, was used. We treated HepG2 cells with 2.5 μmol/L T-AⅢ or 1 μmol/L CITCO for 24 h, and found that T-AⅢ up-regulated CAR just as CITCO (***Fig. 3E***). Additionally, the up-regulation effect of T-AⅢ on CYP2B6, CYP3A4, and MDR1 was similar to that of CITCO. Furthermore, we detected mCAR expression levels in the liver tissues of both nude mice and ICR mice, and found that mCAR expression levels were up-regulated two-fold after 5 and 10 mg/kg body weight T-AⅢ treatment in both types of mice, compared with each control group, respectively (***Fig. 3F*** and ***3G***).

**Figure 4:**
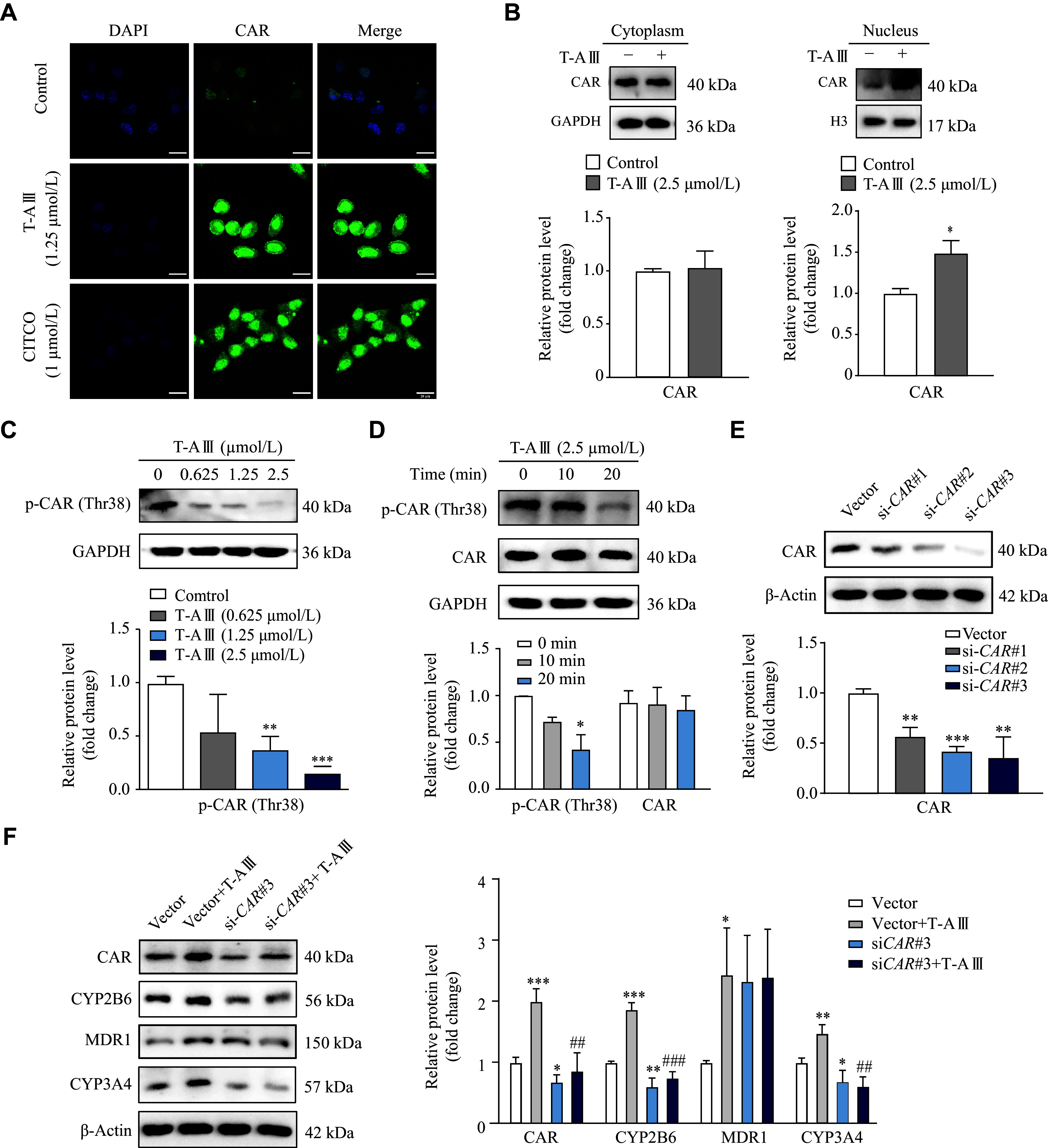
T-AⅢ induced expression levels of CYP2B6 and CYP3A4 by CAR activation.

The activation of CAR was further examined by immunofluorescence assay and nuclei isolation. HepG2 cells were treated with 1.25 μmol/L T-AⅢ and 1 μmol/L CITCO for 12 h, and the immunofluorescence results showed that T-AⅢ significantly increased the fluorescence intensity of CAR, compared with the control, especially in nuclei, similar to that of CITCO (***Fig. 4A***). Furthermore, upon treatment of 2.5 μmol/L T-AⅢ for 12 h, the expressions levels of CAR were significantly increased in the nucleus of HepG2 cells (***Fig. 4B***).

Dephosphorylation of Thr-38 plays a key role in CAR translocation^[23]^. In HepG2 cells treated with 1.25 μmol/L and 2.5 μmol/L T-AⅢ for 24 h, the protein levels of p-CAR (Thr38) were significantly decreased, compared with the control group (***Fig. 4C***). In HepG2 cells treated with 2.5 μmol/L T-AⅢ for 20 min, the protein levels of p-CAR (Thr38) were also significantly decreased, compared with the control group (***Fig. 4D***). These results indicate that T-AⅢ activates CAR, which may be responsible for the up-regulation of its target genes, *i.e.*, *CYP2B6*, *CYP3A4*, and *MDR1*.

For further verification, HepG2 cells were transfected with *CAR* siRNA followed by T-AⅢ (2.5μmol/L) treatment for 24 h. The Western blotting analysis revealed that the *CAR* siRNA transfection significantly decreased CAR expression levels (***Fig. 4E***) and reversed the increased expression levels of CAR, CYP2B6, and CYP3A4 induced by T-AⅢ (***Fig. 4F***). However, *CAR* siRNA did not reverse the increase of MDR1 induced by T-AⅢ in HepG2 cells. These results demonstrate that T-AⅢ induces CYP2B6 and CYP3A4 by CAR activation.

### Effects of T-AⅢ on other CAR target genes

**Figure 5:**
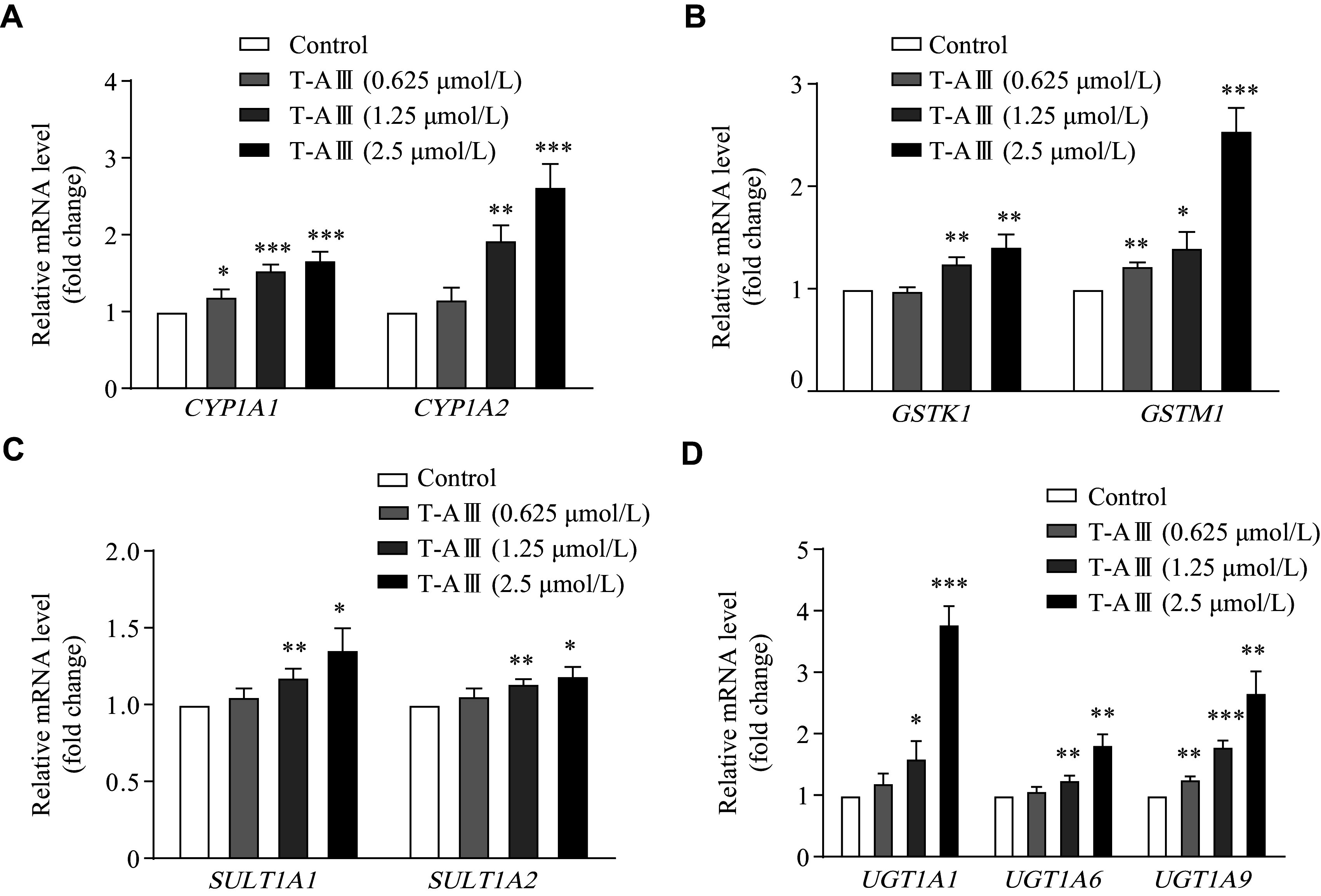
T-AⅢ up-regulated expression levels of the CAR target genes.

Besides CYP2B6 and CYP3A4, the nuclear receptor CAR regulated the transcription of its numerous target genes. To examine the effects of T-AⅢ on these target genes, we analyzed mRNA levels of the phase Ⅰ and phase Ⅱ liver-metabolizing enzymes in HepG2 cells. The results showed that T-AⅢ significantly increased transcriptional levels of *CYP1A1* and *CYP1A2* (***Fig. 5A***), *GSTK1* and *GSTM1* (***Fig. 5B***), *SULT1A1* and *SULT1A2* (***Fig. 5C***), and *UGT1A1*, *UGT1A6* and *UGT1A9* (***Fig. 5D***) in a concentration-dependent manner. These findings indicate that T-AⅢ may enhance the transcription of *CAR* target genes, and further demonstrate that T-AⅢ-mediated the induction and activation of CAR.

### T-AⅢ induced and activated CAR expression in HepG2 cells by inactivating the ERK1/2 signaling pathway

**Figure 6:**
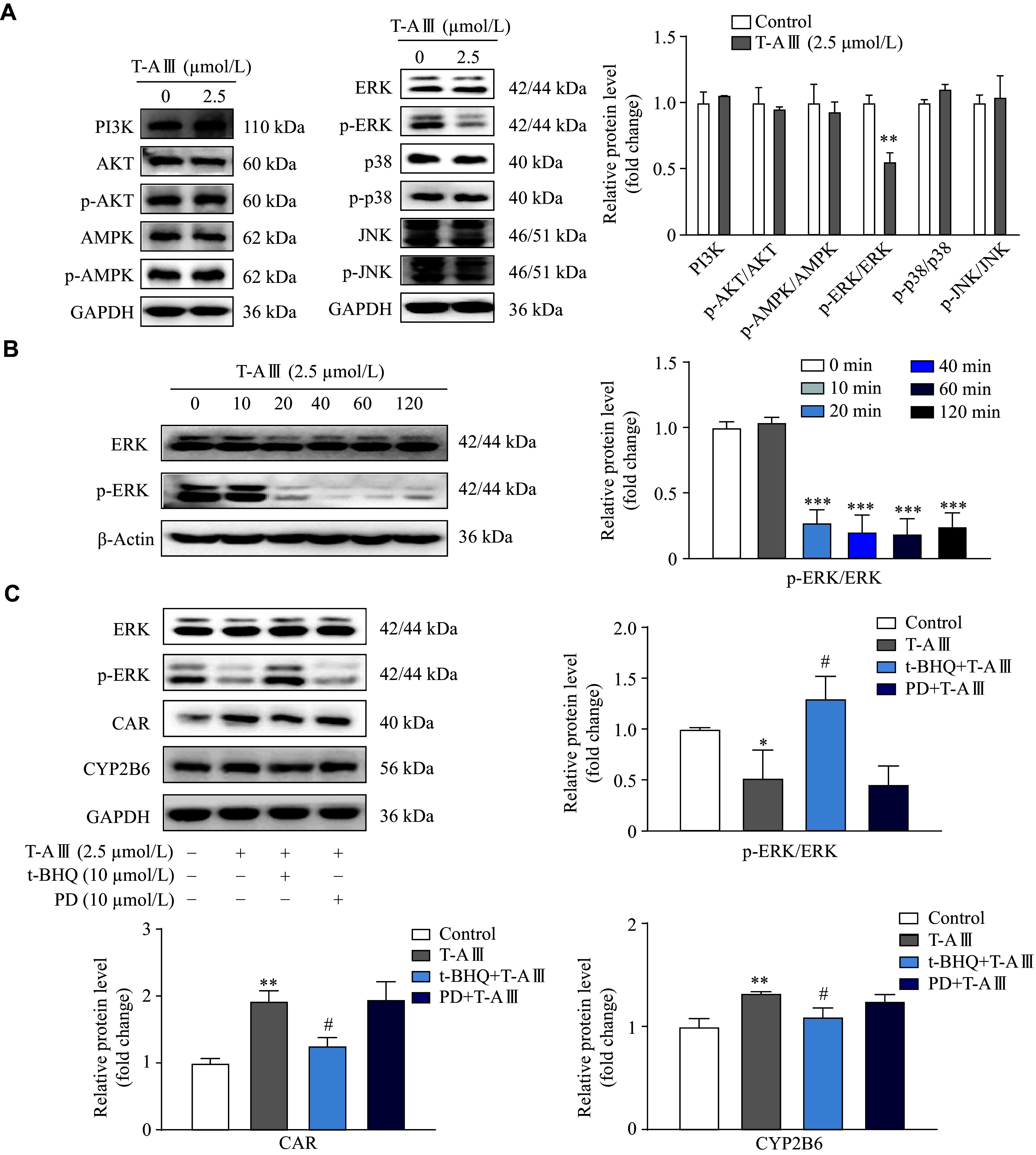
T-AⅢ induced and activated CAR by inactivating the ERK1/2 signaling pathway.

To investigate the mechanism of T-AⅢ-mediated CAR activation, we detected the protein levels of different signaling pathways related to CAR in HepG2 cells. As shown in ***Fig. 6A***, T-AⅢ significantly inhibited the ERK1/2 phosphorylation, while there were no changes observed in the PI3K, AKT, p38, JNK, and AMPK signaling pathways. Additionally, time course results showed significant decreases in ERK1/2 phosphorylation after treatment with 2.5 μmol/L T-AⅢ for 20 min or longer (***Fig. 6B***). It has been demonstrated that the phosphorylation of ERK1/2 prevents the nuclear translocation of CAR in the hepatocytes^[24]^. The evidence indicates that T-AⅢ may mediate the activation of CAR by inhibiting ERK1/2 phosphorylation.

To further investigate the effects of T-AⅢ-mediated suppression of ERK1/2 phosphorylation on the induction and activation of CAR as well as its target genes, HepG2 cells were treated with 10 μmol/L of t-BHQ (ERK activator) or 10 μmol/L of PD98059 (MEK inhibitor) 1 h before T-AⅢ treatment for another 24 h. As shown in ***Fig. 6C***, T-AⅢ significantly inhibited ERK1/2 phosphorylation, which was restored by t-BHQ treatment but remained unaffected by PD98059 treatment. Additionally, T-AⅢ significantly enhanced the expression levels of CAR and CYP2B6, which was partially attenuated by t-BHQ treatment. However, PD98059 did not affect T-AⅢ-mediated induction of CAR and CYP2B6 (***Fig. 6C***). These results indicate that T-AⅢ may induce the expression of CAR and its target genes *via* suppressing ERK1/2 phosphorylation.

**Figure 7:**
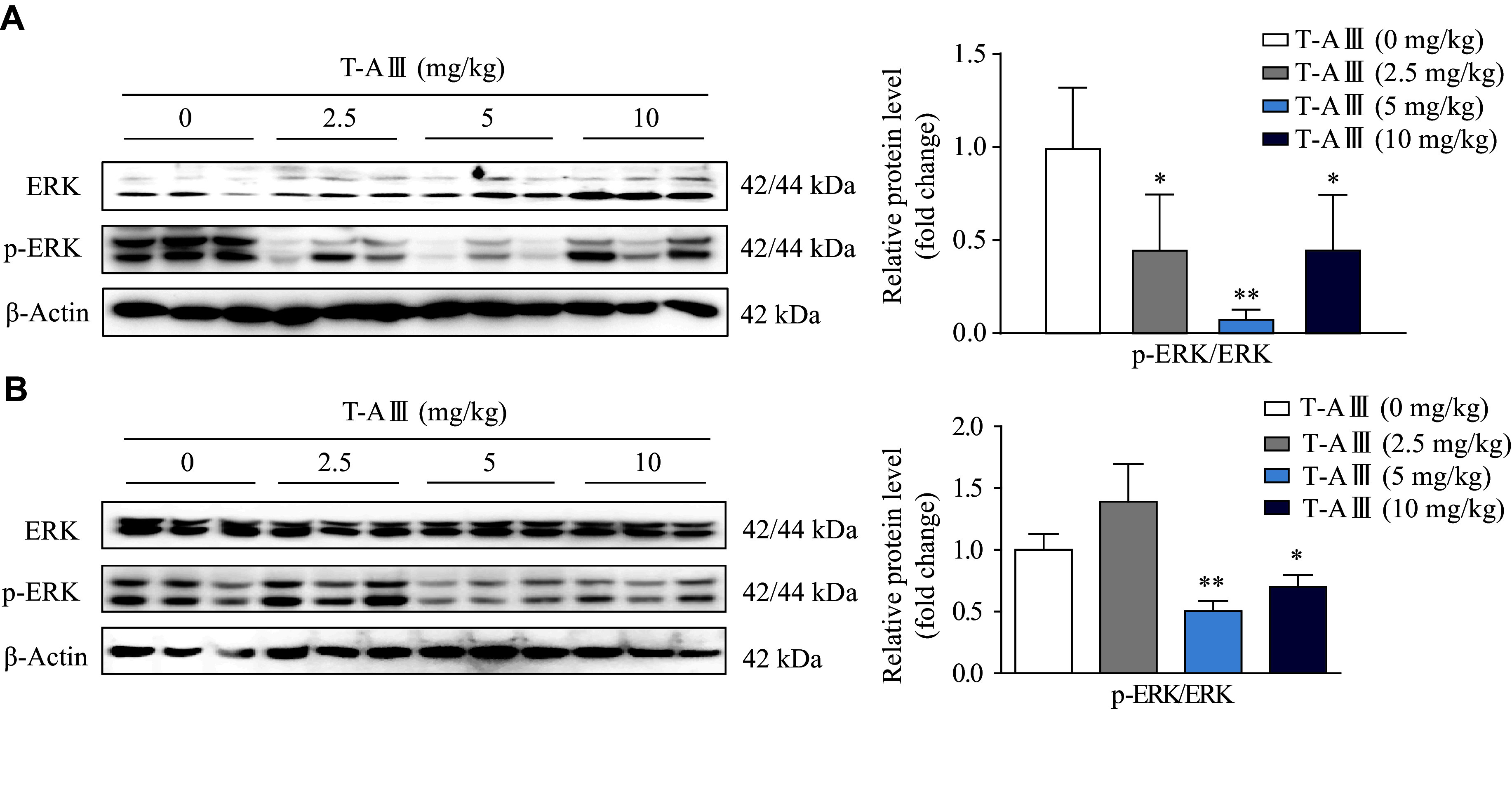
*In vivo* effects of T-AⅢ on the ERK1/2 signaling pathway.

To further validate the effect of T-AⅢ on p-ERK1/2 *in vivo*, we detected p-ERK1/2 expression levels in the liver tissues of both nude and ICR mice. The results showed that p-ERK1/2 was significantly down-regulated after T-AⅢ treatment in both two types of mice (***Fig. 7A*** and ***7B***). These results suggest that T-AⅢ up-regulated mCAR and its target genes (*CYP2B10* and *MDR1*) *in vivo* *via* the inactivation of the ERK1/2 signaling pathway, consistent with the *in vitro* findings.

### T-AⅢ inhibited the phosphorylation of EGFR

**Figure 8:**
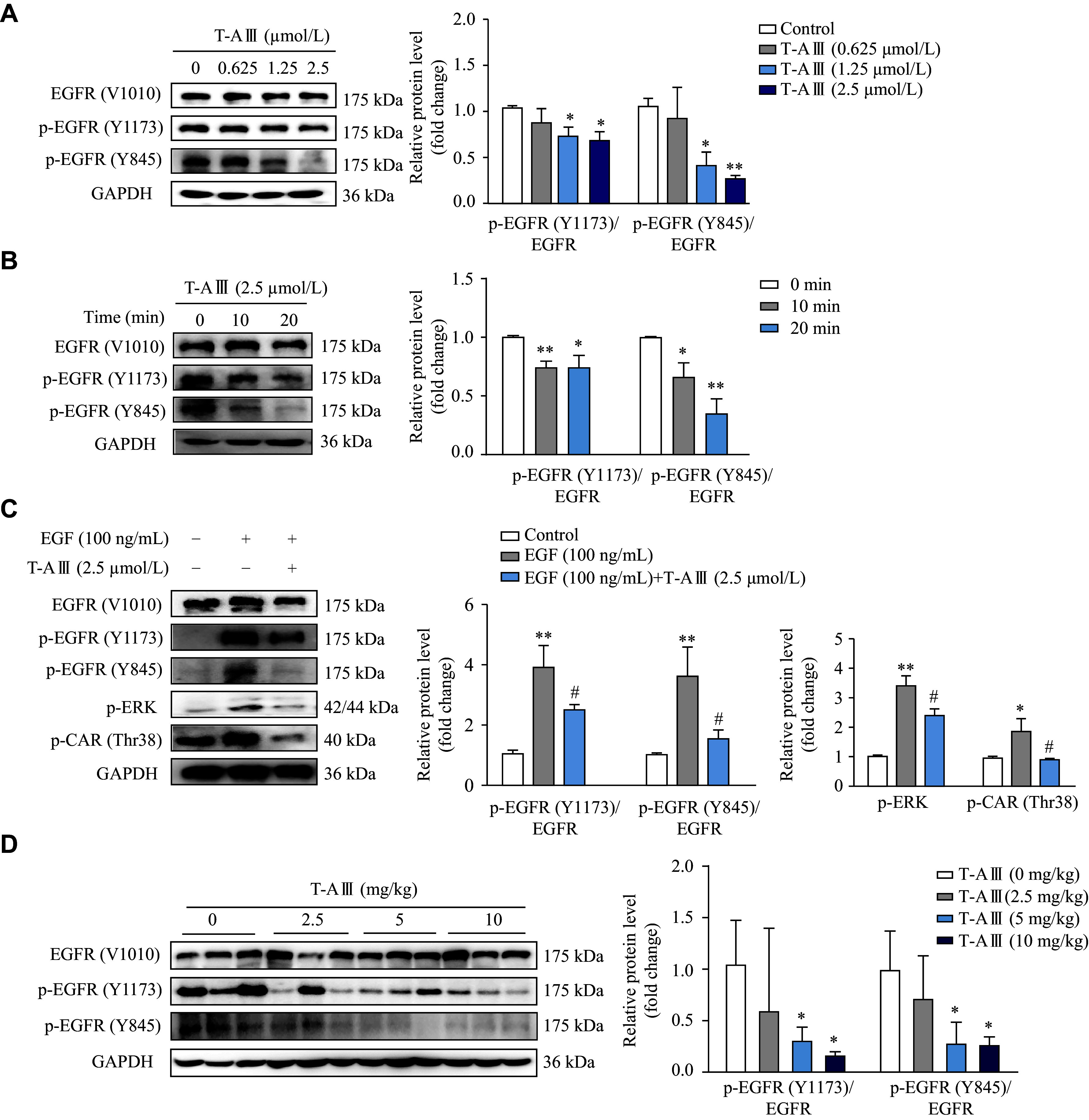
Effect of T-AⅢ on EGFR phosphorylation.

Epidermal growth factor receptor (EGFR) plays a crucial role in the activation of CAR^[21]^. It has been demonstrated that epidermal growth factor (EGF) represses CAR expression by activating the MEK/ERK signaling pathway^[25]^. Therefore, we investigated the effects of T-AⅢ on the activation of EGFR in HepG2 cells. HepG2 cells were treated with various concentrations of T-AⅢ for 24 h, and then EGFR, p-EGFR (Y1173), and p-EGFR (Y845) were detected by Western blotting. As shown in ***Fig. 8A***, T-AⅢ significantly reduced the expression levels of p-EGFR (Y1173) and p-EGFR (Y845) in a concentration-dependent manner. Subsequently, we treated HepG2 cells with 2.5 μmol/L T-AⅢ for various durations, and found that the expression levels of p-EGFR (Y1173) and p-EGFR (Y845) decreased significantly after T-AⅢ treatment for 10 min (***Fig. 8B***).

Furthermore, EGF was used to activate the EGFR signaling pathway. The results showed that T-AⅢ significantly attenuated the expression levels of p-EGFR (Y1173), p-EGFR (Y845), p-ERK, and p-CAR (Thr38) upregulated by EGF treatment (***Fig. 8C***). Subsequently, we investigated the effects of T-AⅢ on EGFR phosphorylation in the liver tissues of nude mice, and found that p-EGFR (Y1173) and p-EGFR (Y845) expression levels were significantly down-regulated by T-AⅢ treatment (***Fig. 8D***). These results indicate that T-AⅢ may inhibit the phosphorylation of EGFR at Tyr^1173^ and Tyr^845^, thereby inactivating the ERK1/2 signaling pathway and suppressing CAR phosphorylation.

## Discussion

Traditional Chinese medicine has been employed for centuries in treating various diseases and is considered a crucial source for new drug discovery. T-AⅢ, an active constituent, has been demonstrated to have significant anti-tumor potential across various cancer cells^[3]^. The reported molecular mechanisms of T-AⅢ encompass the induction of the cell cycle, apoptosis, autophagy, ferroptosis, inhibition of invasion, and angiogenesis. Nevertheless, further investigation is needed to comprehensively understand each aspect to facilitate the clinical use of T-AⅢ as a novel antitumor agent in the near future. Thus, the current study focused on the effects of T-AⅢ on drug-metabolizing enzymes.

Drug-metabolizing enzymes play a pivotal role in drug metabolism, elimination, and detoxification, influencing the efficacy and toxicity of drugs^[26]^. Among these enzymes, CYP3A4 metabolizes more drugs in clinical use than any other metabolizing enzyme in humans, contributing to approximately 50% of all drug metabolism^[27]^. CYP2B6 has been shown to metabolize 2% to 10% of clinically used drugs, including widely used antineoplastic agents such as cyclophosphamide and ifosfamide, anesthetics like propofol and ketamine, synthetic opioids such as pethidine and methadone, and antiretrovirals like nevirapine and efavirenz, among others^[28]^. MDR1 is responsible for multidrug resistance, with numerous anti-cancer agents identified as its substrates, including anthracyclines, taxanes, vinca alkaloids, camptothecins, epipodophyllotoxins, and tyrosine kinase inhibitors^[29]^.

In the current study of the anticancer effects of T-AⅢ in nude mice, we observed that T-AⅢ induced the expression levels of CYP2B10, MDR1, and CYP3A4 in the mouse liver. We also obtained similar results in ICR mice following a short-term administration. Subsequently, in HepG2 cells, T-AⅢ induced the expression of CYP2B6, MDR1, and CYP3A4 at both mRNA and protein levels. These findings suggest that T-AⅢ may influence the metabolism of clinically used substrate drugs by up-regulating the expression of CYP2B6, CYP3A4, and MDR1, thereby affecting the efficacy and toxicity of the drugs. T-AⅢ has been reported to induce multidrug resistance reversal by inhibiting the expression of MDR1 and MRP1 in human chronic myelogenous leukemia K562/ADM cells^[30]^. Additionally, recent findings by Dai *et al*^[31]^ suggest that T-AⅢ may serve as both a substrate and an inhibitor of MDR1 in MDCK-MDR1 cells.

PXR and CAR regulate overlapping sets of genes that encode phase Ⅰ and Ⅱ enzymes, as well as transporters involved in xenobiotic detoxification and elimination. Furthermore, they may also be activated by the same compounds^[32]^. Specifically, PXR and CAR may bind to motifs, such as NR1, NR2, DR3, ER6, DR4, DR5, and gtPBREM, within the promoter regions of their target genes^[17]^. *CYP2B6*, *CYP3A4*, and *MDR1* are target genes of both PXR and CAR. Our results revealed a significant increase in the mRNA and protein levels of CAR after T-AⅢ treatment. In contrast, the expression of PXR remained unchanged (data not shown). Furthermore, T-AⅢ induced the activation of CAR, which was reversed by *CAR* siRNA, resulting in decreased levels of CYP2B6 and CYP3A4 induced by T-AⅢ. These results suggest that T-AⅢ up-regulates the expressions of CYP2B6 and CYP3A4 both *in vitro* and *in vivo* through the activation of CAR.

The activation of CAR may occur through either direct ligand binding or ligand-independent (indirect) mechanisms, both initiating the nuclear translocation of CAR from the cytoplasm^[7]^. As a xenobiotic-responsive modular protein, CAR may be activated or deactivated by binding with agonistic or antagonistic ligands. The discovery of TCPOBOP marked the identification of the first agonist for mouse CAR^[33]^. However, the majority of CAR activators, such as PB^[21]^, do not directly bind to CAR. The dephosphorylation of Thr-38 is pivotal for CAR translocation, irrespective of exposure to direct or indirect activators^[23]^. Several kinase signaling pathways have been implicated in the phosphorylation of CAR^[18]^, including protein phosphatase 2A (PP2A), ERK, p38MAPK, JNK, and AMPK. To determine the molecular mechanism of T-AⅢ-mediated CAR activation, we examined various signaling proteins. Our results revealed a significant inhibition of the ERK signaling pathway by T-AⅢ treatment for 20 min. Additionally, the ERK activator (t-BHQ) partially counteracted the T-AⅢ-induced up-regulation of CAR and CYP2B6. ERK plays a role in CAR phosphorylation, and the activated ERK1/2 interacts with Thr-38 phosphorylated CAR, thereby maintaining CAR phosphorylation and cytoplasmic localization^[25]^. Therefore, our findings indicate that T-AⅢinduces the up-regulation of CAR and its target gene through the deactivation of p-ERK1/2. Furthermore, we also validated these results *in vivo*.

EGFR, a member of the ErbB receptor family, orchestrates extracellular signals, such as EGF, directing cellular signaling cascades to promote cell proliferation, division, mitosis, and cancer development^[34]^. EGFR monoclonal antibodies and small molecule tyrosine kinase inhibitors have achieved good therapeutic effects in clinical cancer treatment^[35]^. Simultaneously, the EGF signaling pathway plays a pivotal role in CAR regulation. In primary human hepatocytes, EGF specifically suppresses the CAR signaling, primarily through transcriptional regulation^[25]^. The crosstalk between CAR and EGFR underscores the interconnectedness of cell proliferation/survival and liver detoxification/metabolism^[21]^. Some evidence indicates that EGF stimulates CAR homodimerization, forcing CAR into its inactive form^[20]^. It has been reported that PB activates CAR transcriptional activity by preventing EGFR phosphorylation^[36]^. PB competitively reverses the EGF signal at the EGFR, triggering the dissociation of ERK1/2 from the CAR homodimer and converting it into a monomer eligible for dephosphorylation at Thr38. Carazo *et al*^[37]^ reported that teriflunomide was an indirect human CAR activator interacting with EGF signaling. The current study data demonstrated that T-AⅢ significantly reduced the expression levels of p-EGFR (Y1173) and p-EGFR (Y845) after a 10-min T-AⅢ treatment, followed by a decrease in p-ERK and p-CAR (Thr38) expression. Furthermore, T-AⅢ inhibited EGF-induced phosphorylation of EGFR, ERK, and CAR. In nude mice, T-AⅢ also inhibited the phosphorylation of EGFR. These results suggest that T-AⅢ induces CAR and its target genes by inhibiting the EGFR signaling pathway, mediated by the dephosphorylation of p-ERK1/2. However, the exact mechanism by which T-AⅢ inhibits EGFR phosphorylation was not elucidated in the current study, and we will continue this investigation in our future work.

Numerous previous studies highlighted the crucial role of CAR in regulating gene expression in response to both exogenous chemicals and endogenous compounds^[7]^. However, it is noteworthy that CAR has been associated with acetaminophen-induced hepatotoxicity^[38]^ and the promotion of liver tumors induced by phenobarbital and TCPOBOP^[39–40]^. Given the role of T-AⅢ as a CAR activator, further investigations are necessary to determine whether T-AⅢ may potentially induce liver toxicity. Notably, in our experiments with both nude and ICR mice, no indications of liver toxicity were observed (data not shown).

The current *in vitro* and *in vivo* analyses have demonstrated that T-AⅢ induces CAR expression, promotes CAR translocation into the nucleus, and subsequently regulates its target genes. Further investigation has revealed that T-AⅢ acts as a novel indirect activator of CAR by inhibiting the EGFR signaling pathway. CAR is implicated in various human diseases, including cancer, liver diseases, inflammatory conditions, metabolic disorders, and diabetes, with a crucial role in drug metabolism and disposition. These findings indicate that T-AⅢ may have the potential to modulate these diseases through the activation of CAR.
